# Dietary Patterns among Vietnamese and Hispanic Immigrant Elementary School Children Participating in an After School Program

**DOI:** 10.3390/nu9050460

**Published:** 2017-05-05

**Authors:** Megan A. McCrory, Charles L. Jaret, Jung Ha Kim, Donald C. Reitzes

**Affiliations:** 1 Department of Health Sciences, Programs in Nutrition, Sargent College of Health and Rehabilitation Sciences, Boston University, Boston, MA 02215, USA; 2 Department of Sociology, College of Arts and Sciences, Georgia State University, Atlanta, GA 30302, USA; cjaret@gsu.edu (C.L.J.); jhkim@gsu.edu (J.H.K.); dreitzes@gsu.edu (D.C.R.)

**Keywords:** food preferences, food habits, diet/standards, gender factors, acculturation

## Abstract

Immigrants in the U.S. may encounter challenges of acculturation, including dietary habits, as they adapt to new surroundings. We examined Vietnamese and Hispanic immigrant children’s American food consumption patterns in a convenience sample of 63 Vietnamese and Hispanic children in grades four to six who were attending an after school program. Children indicated the number of times they consumed each of 54 different American foods in the past week using a food frequency questionnaire. We ranked each food according to frequency of consumption, compared the intake of foods to the USDA Healthy Eating Pattern, and performed dietary pattern analysis. Since the data were not normally distributed we used two nonparametric tests to evaluate statistical significance: the Kruskal–Wallis tested for significant gender and ethnicity differences and the Wilcoxon signed-rank test evaluated the food consumption of children compared with the USDA recommended amounts. We found that among USDA categories, discretionary food was most commonly consumed, followed by fruit. The sample as a whole ate significantly less than the recommended amount of grains, protein foods, and dairy, but met the recommended amount of fruit. Boys ate significantly more grains, proteins, and fruits than did girls. Dietary pattern analysis showed a very high sweet snack consumption among all children, while boys ate more fast food and fruit than girls. Foods most commonly consumed were cereal, apples, oranges, and yogurt. Ethnicity differences in food selection were not significant. The high intake of discretionary/snack foods and fruit, with low intake of grains, vegetables, protein, and dairy in our sample suggests Vietnamese and Hispanic immigrant children may benefit from programs to improve diet quality.

## 1. Introduction

Immigrants in the U.S. may encounter challenges of acculturation, including dietary habits, as they adapt to new surroundings in the host country [1]. Studies show a health paradox in that early in their arrival, immigrants may be healthier than their U.S. counterparts, but later go on to develop health risks as they adapt to a more Western lifestyle [2]. In general, a higher level of acculturation is associated with greater risk for chronic diseases [3,4]. The impact of acculturation may in part be responsible for the large disparities in rates of obesity and chronic disease among U.S. minority immigrants [5]. In the U.S., African Americans and Hispanics are at higher risk for obesity, diabetes [6], and heart disease [7] than Caucasians. Few studies have been conducted among Asian Americans. A recent U.S. national survey showed that although non-Hispanic Asians generally are not as unhealthy as other U.S. adults, there was great diversity in the health of different Asian American groups [8]. In particular, 16.8% of Vietnamese adults were considered to be in fair or poor health, compared to 8.1–12.2% for other Asian American groups and 12.4% for all U.S. adults [8].

Dietary choices during childhood are important for health during childhood and later life [9,10]. Therefore, the dietary intake of immigrant children is important for their health as adults. Relatively few studies have been conducted on the dietary intake of immigrant children. As reviewed recently [11], Asian American youth as a whole have high intakes of fruits, vegetables, and white rice, as well as high fat and high sugar foods, and as a result of acculturation their diets consist of both traditional Asian foods and American foods. “Asian Americans” include many different Asian cultures, each having different diets, but relatively few studies have been done on specific Asian subgroups so it is difficult to make firm conclusions about each. In one study of Vietnamese, Hispanic, African American and Caucasian adolescents residing in Worcester, Massachusetts, Vietnamese youth had higher fruit and vegetable intake and lower dairy intake compared to Caucasians, while Hispanic youth had a lower intake of fruits and vegetables, but dairy did not differ from that of Caucasians [12]. In another study of California youth, Mexican and other Hispanic children did not differ substantially in dietary practices, whereas among seven Asian American subgroups, Filipino, Korean, Vietnamese, and Japanese children were more likely to consume fast food than Chinese children [13]. Yet, Vietnamese, Koreans, and Filipinos were also likely to consume more vegetables than the Chinese. In the same study, there was no significant gender difference in consumption among Hispanic youth, but among Asian Americans, girls had significantly lower vegetable intake, and non-significantly lower fruits, fruit juice, and fast food intake. In a study of Korean Americans, there was a shift away from Korean foods to more American foods, but the quality of diet did not vary by acculturation status [14]. However, a study of South Asian immigrants in Canada showed a mix of positive and negative outcomes: the more acculturated ate more fruits and vegetables and less deep fried food, but also more convenience food, red meat, and high-sugar foods [15].

The purposes of this study were to examine the quality of food selection in a convenience sample of school-aged immigrant Vietnamese and Hispanic girls and boys based on their answers on a simple food intake questionnaire, and to test for differences by gender and ethnicity. We defined the quality of food selection in two ways: (1) consistency with the USDA Healthy Eating Pattern [16]; and (2) dietary pattern analysis to determine which foods listed on the questionnaire were eaten in similar patterns (e.g., if children who ate a lot of chocolate candy also frequently ate cookies and cake; or if those who rarely ate apples also did not eat other fruits).

## 2. Materials and Methods

### 2.1. Study Design

The data used in this study were from a survey, “My Food Choices,” conducted by the Asian American Community Research Institute of the Center for Pan Asian Community Services, Inc. (CPACS) in Atlanta, GA, USA. We obtained the data with permission from CPACS. CPACS is the oldest and largest grassroots community organization in the Southeast serving Asian immigrant and refugee families and their descendants [17]. A considerable number of Hispanic children also participate in its after-school programs. As of December 2015, over 40 percent of all children and youth programs participants at CPACS were Hispanic [18,19].

### 2.2. Participants and Recruitment

The survey was conducted at three of CPACS’s after-school program sites in January and February of 2012. CPACS’s purpose in conducting the survey was to determine the types of foods and beverages readily available and consumed by the children attending its after-school programs. The children participating in these programs were either Vietnamese or Hispanics living in a low-income immigrant community. All children who participated in the survey were fourth, fifth, and sixth graders, eligible for free lunch at school and had at least one immigrant or refugee parent/guardian.

Because the survey targeted young children, CPACS sent a bi-lingual letter to each child’s parents to obtain informed consent for their child’s participation in the survey. Data were collected from only the children with parental approval to participate in the survey. A total of 63 children (approximately 90% of those who were eligible) completed the survey. On-site teachers or tutors recorded site information and assigned a unique identification number to all collected surveys. The present research team obtained the questionnaires in Fall 2015. Because the questionnaires were de-identified, the Institutional Review Board at Georgia State University determined the project was not human subjects research as per U.S. Dept. of Health and Human Services.

### 2.3. Instruments

**Figure A1:**
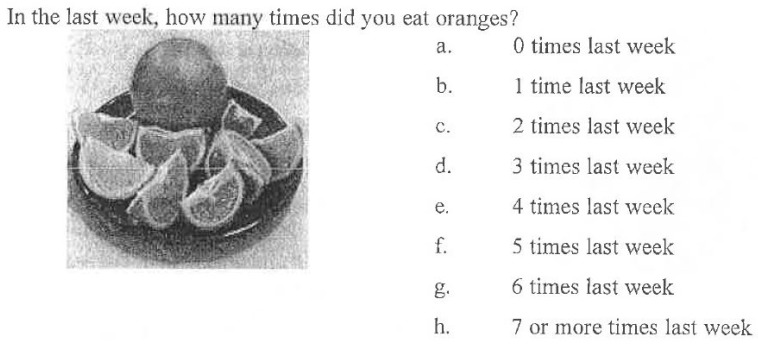
Sample question from 54 item food frequency questionnaire. The full questionnaire is available from the authors upon request.

The “My Food Choices” questionnaire was designed by CPACS staff. The questionnaire consisted of 54 questions, each asking about the frequency of consumption of a type or class of food, e.g., “carrots,” “fried chicken or nuggets,” or “yogurt.” The foods and beverages included on the questionnaire were typical American foods that may be consumed by children at home, school, or restaurants, and represented examples from all food groups (fruits, vegetables, dairy, meat and beans, cereals, sweets). Many of the foods on the questionnaire were often served to the children at CPACS, or in their school cafeteria. A sample question is shown in Appendix A (Figure A1). Next to the name of each food type was a small photo of a serving of the food or a package containing it. For each food type the questionnaire asked: “In the last week, how many times did you eat/drink?”. The possible responses were: zero, one, two, three, four, five, six, and seven or more times. The children read and completed the questionnaire without assistance from CPACS staff or parents. Children took approximately 15–20 min to complete the questionnaire.

### 2.4. Data Analysis

The data were analyzed using SPSS, version 20 (IBM, Armonk, NY, USA). Prior to analysis, a number of steps were taken. First, missing data were filled in when possible using multiple regression analysis and discriminant analysis as described below. Variables were also examined for normality. None of the variables were normally distributed (based on the Shapiro-Wilk statistic and on skewness and kurtosis), therefore the data are described by reporting the median and interquartile range (Q1, Q3), and further analyses were carried out using nonparametric procedures. In addition, while the original 54-item questionnaire had water as one of the food items, this item was excluded from analysis (except to describe consumption frequency) because water, although a required nutrient, does not fit into one of the U.S. Department of Agriculture’s (USDA) food groups [16].

We used the nonparametric Wilcoxon signed-rank test to determine whether there was a statistically significant difference between their actual food consumption and the USDA recommended amounts. Although we were interested in testing for differences by gender within each ethnicity, the small number of Vietnamese (*n* = 15) compared to Hispanic (*n* = 48) children in our sample made it statistically impractical to examine a gender by ethnicity interaction effect. Therefore, our main focus was to test for gender and ethnicity differences. The nonparametric Kruskal–Wallis test was performed to test for differences in food intake between boys and girls. In supplemental analyses, we tested the independent associations of gender and ethnicity with food intake by using ANOVA with gender as a fixed factor and ethnicity as a covariate, modeling only the main effects and not the interaction effects. The results of these analyses were not qualitatively different from those generated by the nonparametric tests. For all analyses, statistical significance was set at α = 0.05.

#### 2.4.1. Response Rate and Procedures for Filling in Missing Data

Overall, children’s response rate on questionnaire was very good, with only 50 of the 3402 potential responses (1.5%) to the food items left unanswered. Where possible, we obtained an estimated value to replace a missing value by utilizing a multiple regression equation to predict how frequently a child ate that food item. A multiple regression model for a food item on which there was missing data was created by using other food items to predict the frequency of consumption of the food item in question. If the best multiple regression model could predict actual consumption of that food item with a high degree of accuracy (i.e., *R*^2^ of 0.60 or higher and low standard error), then it was used to predict the child’s food consumption for that item. If the multiple regression equation was of lessor quality or if the child had more than five missing values on food consumption items, then no estimate was made and a “missing value” code in the data analysis of that food item was retained.

In three cases, data on the child’s gender was missing and in one case the child’s ethnicity was missing. In these cases, discriminant analysis proved highly accurate in distinguishing boys from girls and Vietnamese from Hispanics. Specifically, by identifying a small subset of food items on which consumption levels of boys (or Hispanics) were very different than girls (or Vietnamese), discriminant analysis was able to correctly classify 86.2% of known boys (25 out of 29) boys and 89.7% of known girls (26 out of 29). This discriminant analysis classified two children with missing data on gender as female, while the third case retained a missing value code because it had too much missing data on food consumption items used in the discriminant analysis. Discriminant analysis also correctly classified 83.3% of known Vietnamese children (10 out of 12) and 87.2% of known Hispanic (41 out of 47), and that analysis led to a classification of “Hispanic” for the one case with missing data on ethnicity. Thus, the demographic composition of this sample was: 26 Hispanic boys, 22 Hispanic girls, 5 Vietnamese boys, and 9 Vietnamese girls (one Vietnamese child had missing data for his/her gender).

#### 2.4.2. Determination of Adherence to the USDA Dietary Guidelines for Americans

We categorized each of the food items according to USDA food groups (Table 1). Note that the categorization was not mutually exclusive, i.e., a food could appear in one or more of the groups (e.g., macaroni and cheese was assigned to both the grains and the dairy categories). This is because many foods contain components that belong to different food groups, as described by the USDA [20]. We then compared the actual number of servings per week of vegetables, fruits, grains, dairy, and proteins food groups, as well as oils and discretionary foods consumed by the children with the USDA recommended number of servings for 10-year-old children (which we estimated to be the average age of the children in our sample) based on the USDA Healthy Eating Pattern [16]. To perform this analysis, we assumed that the portion of each food consumed was equivalent to one serving. We calculated the recommended number of servings per week in each food group by multiplying the daily number of recommended servings by seven days per week.

#### 2.4.3. Factor Analysis to Determine Dietary Patterns

We performed factor analysis on the 53 food items (excluding water) with quartimax rotation. We used quartimax rather than varimax rotation because we were more interested in learning which foods load most strongly on a factor (quartimax rotation) than minimizing the number of foods associated with each factor (varimax rotation). Thirteen factors with eigenvalues over 1.00 were produced (accounting for 80.1% of the variance), but the most substantively interesting were the first five factors (which account for 58.1% of total variance). These five factors are shown in Table 2. Factor loadings for food items used to compute each factor score are shown in bold.

## 3. Results

On the first factor (“Veggies Plus”), foods with the highest factor loadings were mainly vegetables (beans, carrots, broccoli, sweet potatoes) plus rice with gravy and three unexpected foods: fish sticks, spaghetti, and pretzels. Children who ranked high on the Veggies Plus factor ate a relatively healthy diet, with low consumption of soda, fruit-flavored drinks, and chips. Factor 2 (“Sweet Snacks”) reflected the least healthy foods, with items like snack cakes, cookies, chips, and ice cream loading strongly on this factor. Children with high scores on the Sweet Snacks factor ate very little greens, peaches, salad, and pears. Factor 3 (“Fruit”) represented fruit consumption, with items like oranges, bananas, apples, and fruit juice having the highest factor scores. Those with high scores on the Fruits factor ate little vegetable soup, salad, or hot wings, and do not drink soda. The foods loading strongest on factor 4 (“Fast Food”) were hamburgers, pizza, hot wings, French fries or tater tots, and fried chicken/nuggets. Those who were high on the Fast Food factor also often ate cheeseburgers and ice cream and infrequently ate fruit, cereal, and vegetables other than corn. The less often eaten vegetables (yellow squash, vegetable soup, tossed salad, and greens) comprised factor 5 (“Other Veggies”), and it partially reflected a healthy diet, since children scoring high on this factor ate little ice cream or cookies, but they also ate little chicken, fruit, and cereal. To produce an index for each of these factors, we summed children’s scores on the food items specified in boldface type in Table 2. The reliability of these indexes is quite good, as Cronbach’s alphas are: factor 1 (vegetables plus) = 0.91; factor 2 (sweets) = 0.91; factor 3 (fruits) = 0.87; factor 4 (fast food) = 0.89; and factor 5 (less popular veggies) = 0.79.

In general, this sample of immigrant children’s food consumption responses clustered at the low end of the range (Table 3). For 17 of the 54 foods listed, 16 had a median of 0 and one had a median of 1, while 20 other items (including carrots, hamburgers, fried chicken or nuggets, and other beans) were eaten only once per week. Cereal was the most often eaten food (its median was four times per week, as was milk for low-fat and whole milk combined). Certain fruits (e.g., apples, oranges, grapes, bananas) were eaten fairly often, but other fruits (pears, peaches, and raisins) were infrequently eaten by most immigrant children. Most protein sources like hamburgers, fried chicken, peanut butter, and chicken were each consumed one or fewer times per week. Concerning beverages, water was most often consumed, followed by fruit-flavored drinks, fruit juice, low fat milk and whole milk, and sodas were consumed the least often. Snack foods like chips, cookies, and ice cream were among the most commonly consumed items at twice per week each, while others of these types of snack foods including snack cakes, cake, pretzels, and honey buns were consumed fairly infrequently.

### 3.1. Consumed vs. Recommended Number of Servings in USDA Food Groups

**Figure 1:**
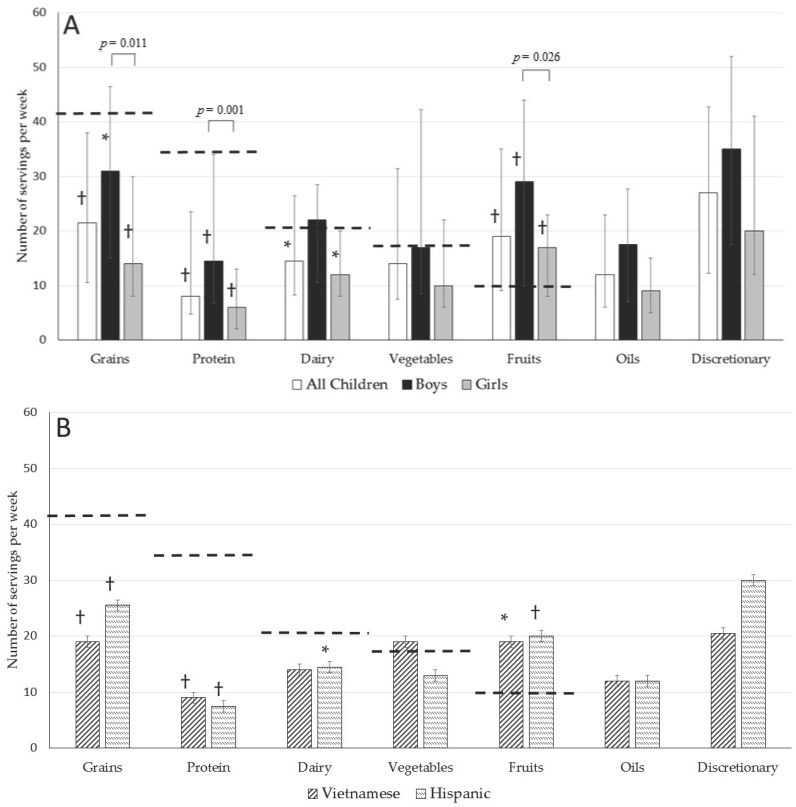
Reported median number of servings per week consumed in USDA food groups versus recommended values in a sample of fourth, fifth, and sixth grade Vietnamese and Hispanic immigrant children (*n* = 63) for (**A**) all children, boys, and girls; and (**B**) Vietnamese and Hispanic children. Error bars show interquartile range (25th and 75th percentiles). Dotted horizontal lines represent USDA recommended number of servings per week for 10-year-old boys and girls [16]. Symbol next to vertical bar indicates significant difference between number of servings consumed compared with the USDA recommendation (* for *p* < 0.05; † for *p* < 0.01). *p*-values are shown for Kruskal–Wallis test for significant differences between boys and girls; *p*-values for gender differences in consumption of other food groups were 0.058 for dairy, 0.117 for vegetables, 0.056 for oils, and 0.09 for discretionary. Differences by ethnicity were not significant (*p*-values ranged from 0.398 to 0.993).

Figure 1 shows the median and interquartile range for consumption per week of foods grouped into USDA categories compared with the recommended number of servings per week, arranged in descending order of the recommendation for all children, boys, girls, Vietnamese, and Hispanic children. The sample as a whole ate significantly less than the recommended amount of grains, protein foods, and dairy, but met the recommended amount of fruit. Examining USDA food group consumption by gender, while the median consumption was higher in boys than in girls for all food groups, the differences were significant for grains, protein foods, and fruits (all *p* < 0.05), and marginally nonsignificant for dairy, oils, and discretionary foods (*p*-values ranged from 0.056 to 0.09). Results were similar when analyzed by ANOVA, controlling for ethnicity (Table S1). In addition, compared with the recommended number of servings, boys’ median consumption was significantly lower for grains and protein foods, and significantly higher for fruits, while girls’ median consumption was significantly lower for grains, protein foods, and dairy, and significantly higher for fruits. For both boys and girls, the highest number of servings came from discretionary foods, followed by grains and fruits. Of the food groups, fruit consumption was relatively high, with 67% of the sample reaching the recommended value (75% of boys and 58% of girls). A relatively high number of boys also reached the recommended number of servings for dairy (58%) and vegetables (50%), while only 31% and 20% of boys reached the recommended number of servings for grains and protein foods, respectively. Only 35% of girls reached the recommended number of servings for vegetables, and 23, 10, and 6% of girls consumed the recommended number of dairy, grain, and protein servings, respectively.

In contrast, the figure also shows that there were no significant differences in food group intake by ethnicity. Results were similar when analyzed by ANOVA, controlling for gender (Table S1). While a majority of Vietnamese and Hispanic children met or exceeded the recommended number of servings of fruits (*p* < 0.05 and *p* < 0.01, respectively), they consumed a significantly lower number of servings of grains and protein foods (*p* < 0.01). In fact, only 8% and 14% of Vietnamese children met or exceeded the recommended amount of grains and proteins, respectively; while only 23% and 13% of Hispanic children ate the recommended amount of grains and protein foods. Most Vietnamese and Hispanic children also consumed a lower number of dairy servings compared with the recommendation, but this difference was significant only for Hispanic children (*p* < 0.05). The recommended number of vegetable servings was met by 62% of Vietnamese but only 38% of Hispanic children.

### 3.2. Dietary Patterns Discerned by Factor Analysis

Dietary patterns from factor analysis for all of the children in our sample, as well as by ethnicity and gender, are shown in Table 4. For all children, foods in the Sweet Snacks and Fruits factors were most frequently consumed, followed by foods in the Veggies Plus factor, then the Fast Food and Other Veggies Factors. Dietary patterns did not differ significantly by ethnicity, although Hispanic children consumed foods in Sweet Snacks much more frequently and Other Veggies much less frequently than did Vietnamese children. When examined by gender, however, frequency of consumption in each factor was higher for boys than for girls. This gender difference reached statistical significance for Fruits and Fast Food factors, and was marginally nonsignificant for the Veggies Plus factor.

## 4. Discussion

We examined dietary patterns based on USDA food groups and dietary pattern analysis (factor analysis) in a convenience sample of Vietnamese and Hispanic school-aged immigrant children attending after-school programs. Several findings are especially interesting. On the positive side, most children in this sample ate the recommended number of servings per week of fruit, and approximately half of the boys ate the recommended number of servings of vegetables and dairy foods. Also, given the concern that many children drink too much soda, another positive finding was that the frequency of soda consumption was low (median was only once per week and interquartile range was 0 to 3). On the negative side, many of the children in our sample reported that for several food groups, their diets were below the recommended number of servings. Indeed, 87% of the sample was below the weekly amount in protein foods, 80% below in grains, 60% below in dairy, and 57% below the recommended weekly servings of vegetables. In addition, we found high levels of consumption of sweet snacks and fast food. Differences between the two ethnicities were not significant, but boys consumed significantly more servings per week of grains, protein, and fruits than girls. These findings suggest a generally unhealthy diet in these immigrant children, and that they may benefit from programs and interventions to improve diet quality.

Our finding of relatively high fruit consumption and low consumption of vegetables and diary is consistent with previous studies that find immigrants’ adoption of American diets and eating patterns is typically not a wholly healthy change [21,22,23] and it may put them at risk of unhealthy outcomes [24,25,26]. In particular, our results in Vietnamese children which showed high consumption frequencies of fruit and vegetables and a low consumption frequency of dairy are in general agreement with previous studies [12,13,27], but there is some disagreement on consumption of meat (which falls into the protein foods group) between our study and another [27]. In addition, the findings of previous studies in Hispanic children with regard to these food groups are mixed [12,13], making comparisons with our results difficult and also mixed. Both ethnicities in our study had a relatively high intake of discretionary foods, which was not high in Vietnamese children in previous research [12] but was high in another study in Hispanic children [13]. Comparison of our data with previous studies must be done with caution due to methodological variations across the studies including differences in sample size, age groups, family income, acculturation status, and secular trends in U.S. food supply since the data across studies were collected from 1986 to 2013. Potentially, the generally unhealthy diet of children in our sample might be attributable to low family incomes (given that all the children qualified for free school lunches) and/or that traditional Vietnamese and Hispanic diets may be lower in protein and dairy foods than Western diets. We note that this survey was not a full inventory of all American foods eaten (nor did it cover traditional Vietnamese or Latin American foods), so it did not include some potential sources of protein (e.g., eggs, pork, or fish other than fish sticks). Therefore, it is possible that the low intake of protein foods is not as severe as these data imply. Nonetheless, the apparent low consumption of protein and other healthy foods found here merits further research. Taken together, our findings highlight the need for extensive investigation of the dietary practices of immigrant groups in general and children in particular. We need to discover whether continued eating of traditional foods by immigrants or their children can alleviate deficits in consumption of healthy food categories, or if they can shift to healthier choices of American foods. Also, we need to investigate how low income, the spatial location of good grocery stores, and the cultural meanings of traditional and American foods affect immigrants’ food choices.

Further, we found interesting differences in food consumption by gender. For most food groups, especially proteins, grains, and fruits, boys reported more servings than girls. Several factors may account for this gender difference in food consumption. First, although within this age group, the recommedations for energy intake and the recommended number of servings in each food group do not differ between boys and girls [16], in US national survey data, boys report a higher energy intake than girls [28]. Therefore, it may be expected that the boys in our sample would report consuming more servings of any or all of the food groups than girls. In addition, there may be some behavioral and cultural factors that potentially contributed to the gender differences in intake observed in our study. Specifically, here may be traditional cultural gender norms that favor boys and place higher value on the good health of boys and men over girls and women [29]. In addition, studies show that immigrant parents allow sons more freedom to explore and adopt American cultural behaviors but often discourage or prohibit it for daughters, preferring that they maintain traditional customs [30,31]. This could help explain the more frequent consumption of American food by boys in our sample. It is also possible that gender norms encourage boys to be more assertive in interpersonal interactions [32] and therefore encourage boys to ask for and expect more servings of food than girls. Similarly, greater food consumption may be perceived as more masculine and as a means of confirming a male masculine identity, as well as a sense of personal empowerment [33]. In stark contrast, traditional gender norms may highlight petite body images for girls and associate positive self-meanings to girls who eat less and are slim in appearance [34,35]. The gender difference in our study may suggest the need for more in-depth study of the relationship between gender norms and the gender-related meanings assigned to food, both within Western culture and the traditional cultures of immigrant groups.

There are several limitations to this study. Beginning with the sample, the respondents were not randomly selected. However, we took advantage of the data which had been collected by CPACS as an opportunity to quantify the diet quality of immigrant children, a population whose diet and health have been understudied. A larger number of Vietnamese respondents would have enabled us to more directly investigate ethnic and cultural differences in food selection. In addition, about 10% of the children who were eligible to complete the survey did not participate, and there could have been differences between children who participated and those who did not, including age, grade level, and family income differences. Concerning the latter, however, any income differences were small, and unlikely to have created bias in the sample since all children were from families whose income was below the U.S. poverty line. Similarly, a question on the survey asking the respondent’s age would have allowed us to control for age differences in our analysis of gender and independently investigate possible age effects. Another limitation is that while we have data on the number of times a child ate each pictured food, we do not know the actual amount of food consumed per serving. Further, the food items pictured in the questionnaire were all typically American and did not include traditional ethnic foods. The 54 foods included on the questionnaire were a subset of all of the actual (or potential) foods that the children in this sample may eat. Although many of the foods listed on the questionnaire were served to the children in their after school programs at CPACS and thus were believed to have been appropriately included, it is possible that a questionnaire containing a longer or different list of foods could have produced results that differed from our findings. In addition, the food consumption data were self-reported (rather than a precise measurement of the volume of food eaten) and therefore are subject to social desirability bias. The study was conducted in January and February, and did not take into account potential effects of seasonality on the results. The study is also limited by its lack of data on children’s families and other demographic information. While all the children in this study were eligible for free lunch at school and had at least one parent/guardian who is an immigrant or refugee, a more complete analysis would have included variables such as total family income, English-speaking ability, parents’ and children’s immigration status, and their length of time in the United States. These limitations mean that we must be cautious in drawing conclusions from our findings. Nevertheless, this research may be a useful initial step in learning about the nutritional status of these two important groups of immigrant children, and can inform future, more in-depth studies on this topic.

## 5. Conclusions

While our sample size was relatively small and non-random, the results of the present study suggest that further study of the diets and food consumption of immigrant and refugee children is an important direction for continued research. First and foremost, the link between food consumption and health outcomes needs to be directly investigated with more comprehensive measures of the children’s diets, health status, and health risks. Further studies should also compare children from different racial and ethnic groups, as well as the possible independent effects of immigration status and social background factors on food selection and health outcomes. The results also may have implications for helping Hispanic, Vietnamese, and other immigrant children. Educational and outreach programs in partnership with community organizations and religious institutions should focus on encouraging families to serve healthful traditional foods, especially those that increase portions of grains and proteins. In this regard, the food consumption of boys and girls may need to be recognized as different with special efforts to offer girls more servings of nutritious foods, and for boys to moderate their consumption of discretionary food such as candy and other high calorie snacks.

## Figures and Tables

**Table 1:** Classification of food frequency questionnaire items into USDA food groups.

Food Group	Food Items
Fruits	fruit juice, bananas, apples, grapes, pears, oranges, raisins, mixed fruit, peaches
Vegetables	green beans, other beans, carrots, greens, broccoli, sweet potatoes, French fries or tater tots, other potatoes, corn, tossed salad, yellow squash, tomatoes, vegetable soup
Grains	cereal, honey buns, pretzels, spaghetti, macaroni and cheese, fried rice, other rice, rice and gravy, hamburger, pizza, cookies, snack cake, cake
Protein Foods	peanut butter, hot chicken wings, chicken not fried, fried chicken or chicken nuggets, fish sticks, hamburger, cheese-burger, pizza
Dairy	low fat milk, whole milk, yogurt, cheese, macaroni and cheese, cheeseburger, pizza, ice cream
Oils	chips, hot chicken wings, fried chicken or chicken nuggets, fish sticks, fried rice, French fries or tater tots, salad, mayonnaise
Discretionary	fruit-flavored drinks, soda, cereal, honey buns, chips, yogurt, rice and gravy, mayonnaise, ice cream, cookies, snack cake, chocolate candy, cake, jam, jelly or syrup

**Table 2:** Dietary patterns based on factor analysis of Vietnamese and Latino children’s food selections (*n* = 63).

	Veggies Plus	Sweet Snacks	Fruit	Fast Food	Other Veggies
fish sticks	**0.797**	0.162	0.050	0.094	−0.072
broccoli	**0.784**	0.112	0.124	0.062	0.228
carrots	**0.770**	0.081	0.309	0.138	−0.002
other beans	**0.747**	0.044	0.145	0.077	−0.027
green beans	**0.709**	0.136	−0.069	0.046	0.158
sweet potatoes	**0.681**	0.212	−0.091	0.181	0.321
rice & gravy	**0.657**	0.249	−0.143	0.198	−0.039
pretzels	**0.653**	0.087	0.437	0.135	0.107
spaghetti	**0.599**	0.335	0.147	0.171	0.117
snack cakes	0.169	**0.824**	0.102	0.199	−0.032
cookies	0.292	**0.789**	0.220	0.087	−0.113
mayonnaise	0.163	**0.781**	0.034	0.056	0.067
chocolate candy	0.102	**0.768**	0.117	0.280	0.003
ice cream	0.184	**0.734**	0.120	0.373	−0.145
cake	0.178	**0.653**	0.139	0.091	0.364
jam, jelly, syrup	−0.022	**0.647**	0.141	−0.108	0.325
chips	−0.050	**0.625**	0.270	0.199	0.034
popcorn	0.210	**0.584**	0.115	0.169	0.060
fruit-flavored drink	−0.103	**0.570**	0.034	0.231	−0.129
oranges	0.016	0.221	**0.816**	0.107	0.030
apples	0.156	0.363	**0.728**	0.075	0.001
bananas	0.217	0.419	**0.726**	0.070	0.063
fruit juice	0.236	0.004	**0.622**	−0.133	−0.202
grapes	0.360	0.340	**0.589**	−0.054	−0.052
peaches	0.522	−0.062	**0.575**	0.283	−0.055
hamburgers	0.216	0.303	0.142	**0.816**	0.064
pizza	0.253	0.371	0.049	**0.782**	−0.013
hot wings	0.284	0.296	−0.005	**0.701**	−0.112
french fries/tater tots	0.216	0.165	−0.013	**0.680**	−0.036
fried chicken/nuggets	0.196	0.355	0.125	**0.641**	0.114
yellow squash	0.374	0.226	0.036	0.002	**0.761**
tomatoes	0.341	0.020	−0.003	0.008	**0.711**
tossed salad	0.208	−0.010	−0.104	−0.004	**0.618**
greens	0.502	−0.081	−0.001	−0.081	**0.350**
Eigenvalue	15.86	5.60	3.82	3.09	2.40
%Variance	29.93	10.57	7.21	5.83	4.53

The factor loadings used to compute each factor score are shown in boldface type.

**Table 3:** Food consumption frequency (per week) for a sample of Vietnamese and Hispanic immigrant children (*n* = 63).

Food	Median (Times/Week)	Interquartile Range	Type of Food	Median (Times/Week)	Interquartile Range
water	>6	4–7	popcorn	1.0	0–3
cereal	4.0	2–7	fried chicken or nuggets	1.0	0–3
apples	3.0	2–7	hot chicken wings	1.0	0–3
oranges	3.0	2–5	peanut butter	1.0	0–3
yogurt	3.0	0–5	macaroni & cheese	1.0	0–3
grapes	2.0	1–6	other beans	1.0	0–3
bananas	2.0	1–5	fried rice	1.0	0–3
chips	2.0	1–5	other rice	1.0	0–3
fruit-flavored drinks	2.0	1–5	cheese	1.0	0–2
pizza	2.0	1–4	other potatoes	1.0	0–2
cookies	2.0	0–5	mayonnaise	0.5	0–3
fruit juice	2.0	0–4	peaches	0.0	0–4
ice cream	2.0	0–5	greens	0.0	0–2
low fat milk	2.0	0–4	cheeseburger	0.0	0–2
mixed fruit	2.0	0–5	green beans	0.0	0–3
broccoli	2.0	0–4	pretzels	0.0	0–2
whole milk	2.0	0–4	jam, jelly, or syrup	0.0	0–2
chocolate candy	1.0	0–5	chicken not fried	0.0	0–2
French fries or tater tots	1.0	0–4	honey buns	0.0	0–2
carrots	1.0	0–4	tomatoes	0.0	0–2
hamburgers	1.0	0–3	tossed salad	0.0	0–2
snack cakes	1.0	0–4	raisins	0.0	0–1
spaghetti	1.0	0–4	sweet potatoes	0.0	0–1
soda	1.0	0–3	vegetable soup	0.0	0–2
pears	1.0	0–3	fish sticks	0.0	0–1
corn	1.0	0–3	yellow squash	0.0	0–1
cake	1.0	0–3	rice with gravy	0.0	0–1

**Table 4:** Median (Q1, Q3) consumption frequency per week of factor analysis-derived dietary patterns for a sample of Vietnamese and Hispanic immigrant children (*n* = 63).

	All Children	Ethnicity Analysis			Gender Analysis		
		Vietnamese (*n* = 15)	Hispanic (*n* = 48)	Kruskal–Wallis *p*-Value ^1^	Boys (*n* = 31)	Girls (*n* = 31)	Kruskal–Wallis *p*-Value ^1^
Sweet Snacks	19.0 (7, 34)	12.5 (7, 20)	22.0 (7, 39)	0.160	23.0 (8, 39)	13.0 (7, 26)	0.157
Fruits	15.0 (8, 26)	13.0 (7, 22)	15.5 (8, 29)	0.262	21.0 (9, 33)	13.0 (7, 22)	0.043
Veggies Plus	7.0 (4, 20)	7.0 (4, 13)	7.5 (4, 21)	0.951	11.0 (5, 37)	6.0 (3, 15)	0.069
Fast Food	6.0 (3, 16)	8.0 (3, 15)	6.0 (2, 21)	0.853	9.5 (4, 23)	4.0 (2, 14)	0.029
Other Veggies	2.0 (0, 8)	7.5 (1, 10)	2.0 (0, 7)	0.185	2.0 (0, 14)	2.0 (0, 8)	0.648

^1^ Probabilities are statistical significance of Kruskal–Wallis test for differences in consumption of foods in each factor by ethnicity and gender. One Vietnamese child whose gender was unknown was not included in the gender analysis.

## Supplementary Materials

The following are available online at www.mdpi.com/2072-6643/9/5/460/s1, Table S1: Independent associations of gender and ethnicity with USDA food groups and dietary patterns from factor analysis in a sample of Vietnamese and Hispanic children (*n* = 63).

## References

[1] Wang Y., Min J., Harris K., Khuri J., Anderson L.M. (2016). A systematic examination of food intake and adaptation to the food environment by refugees settled in the United States. Adv. Nutr..

[2] Rosas L.G., Guendelman S., Harley K., Fernald L.C., Neufeld L., Mejia F., Eskenazi B. (2011). Factors associated with overweight and obesity among children of Mexican descent: Results of a binational study. J. Immigr. Minor. Health.

[3] Kobel S., Lammle C., Wartha O., Kesztyus D., Wirt T., Steinacker J.M. (2017). Effects of a randomised controlled school-based health promotion intervention on obesity related behavioural outcomes of children with migration background. J. Immigr. Minor. Health.

[4] Wang S., Quan J., Kanaya A.M., Fernandez A. (2011). Asian Americans and obesity in California: A protective effect of biculturalism. J. Immigr. Minor. Health.

[5] Wang M.C., Yoo G.J., Le M.-N., Oda A.Y. (2013). Obesity and Asian Americans: Prevalence, risk factors, and future research directions. Handbook of Asian American Health.

[6] Goran M.I., Ball G.D., Cruz M.L. (2003). Obesity and risk of type 2 diabetes and cardiovascular disease in children and adolescents. J. Clin. Endocrinol. Metab..

[7] Chen J.L., Weiss S., Heyman M.B., Lustig R. (2011). Risk factors for obesity and high blood pressure in Chinese American children: Maternal acculturation and children's food choices. J. Immigr. Minor. Health.

[8] Bloom B., Black L.I. (2016). Health of non-Hispanic Asian adults: United States, 2010–2014. NCHS Data Brief.

[9] Kelder S.H., Perry C.L., Klepp K.I., Lytle L.L. (1994). Longitudinal tracking of adolescent smoking, physical activity, and food choice behaviors. Am. J. Public Health.

[10] Qi Y., Niu J. (2015). Does childhood nutrition predict health outcomes during adulthood? Evidence from a population-based study in China. J. Biosoc. Sci..

[11] Diep C.S., Foster M.J., McKyer E.L., Goodson P., Guidry J.J., Liew J. (2015). What are Asian-American youth consuming? A systematic literature review. J. Immigr. Minor. Health.

[12] Wiecha J.M., Fink A.K., Wiecha J., Hebert J. (2001). Differences in dietary patterns of Vietnamese, White, African-American, and Hispanic adolescents in Worcester, Mass. J. Am. Diet. Assoc..

[13] Guerrero A.D., Ponce N.A., Chung P.J. (2015). Obesogenic dietary practices of Latino and Asian subgroups of children in California: An analysis of the California Health Interview Survey, 2007–2012. Am. J. Public Health.

[14] Lee S.K., Sobal J., Frongillo E.A. (1999). Acculturation and dietary practices among Korean Americans. J. Am. Diet. Assoc..

[15] Lesser I.A., Gasevic D., Lear S.A. (2014). The association between acculturation and dietary patterns of South Asian immigrants. PLoS ONE.

[16] U.S. Department of Health and Human Services, U.S. Department of Agriculture (2015). 2015–2020 Dietary Guidelines for Americans.

[17] Center for Pan Asian Community Services. http://www.cpacs.org.

[18] Center for Pan Asian Community Services (2014). 2013–2014 Annual Report.

[19] Center for Pan Asian Community Services (2015). 2014–2015 Annual Report.

[20] Bowman S.A., Clemens J.C., Friday J.E., Theorig R.C., Mosfegh A.J. (2014). Food Patterns Equivalents Database 2011–12: Methodology and User Guide.

[21] Creighton M.J., Goldman N., Pebley A.R., Chung C.Y. (2012). Durational and generational differences in Mexican immigrant obesity: Is acculturation the explanation?. Soc. Sci. Med..

[22] Lara M., Gamboa C., Kahramanian M.I., Morales L.S., Bautista D.E. (2005). Acculturation and Latino health in the United States: A review of the literature and its sociopolitical context. Annu. Rev. Public Health.

[23] Park S.Y., Murphy S.P., Sharma S., Kolonel L.N. (2005). Dietary intakes and health-related behaviours of Korean American women born in the USA and Korea: The Multiethnic Cohort Study. Public Health Nutr..

[24] Cho Y., Frisbie W.P., Hummer R.A., Rogers R.G. (2004). Nativity, duration of residence, and health of Hispanic adults in the United States. Int. J. Migr. Rev..

[25] Jasti S., Lee C.H., Doak C. (2011). Gender, acculturation, food patterns, and overweight in Korean immigrants. Am. J. Health Behav..

[26] Yang E.J., Chung H.K., Kim W.Y., Bianchi L., Song W.O. (2007). Chronic diseases and dietary changes in relation to Korean Americans’ length of residence in the United States. J. Am. Diet. Assoc..

[27] Betts N.M., Weidenbenner A. (1986). Dietary intakes, iron status, and growth status of Southeast Asian refugee children. Nutr. Res..

[28] U.S. Department of Agriculture, Agricultural Research Service (2016). What We Eat in America, NHANES 2013–2014, Individuals 2 Years and over (Excluding Breastfed Children), Day 1.

[29] Vlassoff C. (2007). Gender differences in determinants and consequences of health and illness. J. Health Popul. Nutr..

[30] Espiritu Y.L. (2003). We Don’t Sleep around Like White Girls Do: Family, Culture, and Gender in Filipina American Lives.

[31] Zhou M., Bankston C.L. (1998). Growing Up American: How Vietnamese Children Adapt to Life in the United States.

[32] Costa P.T., Terracciano A., McCrae R.R. (2001). Gender differences in personality traits across cultures: Robust and surprising findings. J. Personal. Soc. Psychol..

[33] Turner K., Ferguson S., Craig J., Jeffries A., Beaton S. (2013). Gendered identity negotiations through food consumption. Young Consum..

[34] Wardle J., Robb K.A., Johnson F., Griffith J., Brunner E., Power C., Tovee M. (2004). Socioeconomic variation in attitudes to eating and weight in female adolescents. Health Psychol..

[35] McGinnis J.M., Gootman J.A., Kraak V.I. (2006). Food Marketing to Children and Youth: Threat or Opportunity.

