# Supplementary material for: Dietary Patterns among Vietnamese and Hispanic Immigrant Elementary School Children Participating in an After School Program
Source: Nutrients. 2017 May 5;9(5):460. doi: 10.3390/nu9050460 (PMC5452190; doi:10.3390/nu9050460)
Supplement: Supplementary file 1 [file nutrients-09-00460-s001.pdf]

Table S1. Weekly consumption frequency and independent associations of gender and ethnicity with USDA food groups and dietary patterns from factor analysis in a sample of immigrant children (*n* = 63).

	USDA Food Groups								Dietary Patterns from Factor Analysis			
	**Fruit**	**Vegetables**	**Protein**	**Grains**	**Dairy**	**Oils**	**Discretionary**	**Sweet Snacks**	**Fruit**	**Veggies Plus**	**Fast Food**	**Other Veggies**
Vietnamese [1]	20.3	23.1	14.1	21.2	19.2	12.8	22.1	14.2	14.5	13.5	9.2	6.1
	(3.7)	(4.8)	(3.6)	(3.8)	(3.3)	(2.2)	(3.7)	(2.6)	(2.5)	(4.8)	(1.8)	(1.3)
Hispanic [1]	24.4	20.3	14.4	27.2	17.2	15.0	34.1	24.8	18.9	14.6	10.8	4.8
	(2.4)	(2.8)	(1.9)	(2.8)	(1.7)	(1.7)	(3.4)	(2.8)	(1.7)	(2.2)	(1.6)	(1.0)
p-value [2]	0.604	0.409	0.869	0.670	0.360	0.850	0.179	0.145	0.342	0.945	0.965	0.230
Eta^2 for ethnicity [3]	0.004	0.011	0.000	0.003	0.014	0.001	0.030	0.035	0.014	0.000	0.000	0.023
Boys [1]	28.3	25.2	19.1	32.5	20.1	17.7	36.8	26.5	21.0	17.4	13.6	6.7
	(3.1)	(3.9)	(2.5)	(3.6)	(2.0)	(2.3)	(4.3)	(3.7)	(2.2)	(3.0)	(2.1)	(1.6)
Girls [1]	18.6	16.7	9.2	20.0	15.3	11.4	27.0	19.1	14.8	11.3	7.4	3.8
	(2.6)	(2.8)	(1.9)	(2.7)	(2.2)	(1.5)	(3.6)	(3.0)	(1.8)	(2.6)	(1.3)	(0.7)
p-value	0.026	0.062	0.002	0.013	0.079	0.030	0.170	0.239	0.056	0.131	0.019	0.056
Eta^2 for gender	0.080	0.058	0.147	0.101	0.053	0.079	0.031	0.023	0.059	0.038	0.090	0.061

[1] Values for Vietnamese, Hispanic, boys and girls are Mean (SEM) weekly food consumption frequency.

[2] *p*-values show significance level for main effects of gender and ethnicity based on general linear model univariate analysis of variance with sex as a fixed factor and ethnicity as covariate.

[3] Eta^2 is the proportion of variation explained by the independent variables (ethnicity and gender).
